# Advances and challenges in the prevention and treatment of COVID-19

**DOI:** 10.7150/ijms.47836

**Published:** 2020-07-09

**Authors:** Yan-Jie Han, Zhi-Guang Ren, Xin-Xin Li, Ji-Liang Yan, Chun-Yan Ma, Dong-Dong Wu, Xin-Ying Ji

**Affiliations:** 1 Kaifeng Key Laboratory for Infectious Diseases and Biosafety, School of Basic Medical Science, Henan University, Kaifeng, Henan 475004, China.; 2 Henan International Joint Laboratory of Nuclear Protein Regulation, School of Basic Medical Science, Henan University, Kaifeng, Henan 475004, China.; 3 Clinical Laboratory and Functional Laboratory, Kaifeng Central Hospital, Kaifeng, Henan 475000, China.; 4 School of Stomatology, Henan University, Kaifeng, Henan 475004, China.

**Keywords:** SARS-CoV-2, COVID-19, virological characteristics, clinical treatment, drugs.

## Abstract

Since the end of 2019, a new type of coronavirus pneumonia (COVID-19) caused by the Severe Acute Respiratory Syndrome Coronavirus-2 (SARS-CoV-2) has been spreading rapidly throughout the world. Previously, there were two outbreaks of severe coronavirus caused by different coronaviruses worldwide, namely Severe Acute Respiratory Syndrome Coronavirus (SARS-CoV) and the Middle East Respiratory Syndrome Coronavirus (MERS-CoV). This article introduced the origin, virological characteristics and epidemiological overview of SARS-CoV-2, reviewed the currently known drugs that may prevent and treat coronavirus, explained the characteristics of the new coronavirus and provided novel information for the prevention and treatment of COVID-19.

## Introduction

The spread of COVID-19, which first appeared in December 2019, is still increasing rapidly.^1^,^2^ At present, cases of COVID-19 caused by SARS-CoV-2 have been found in many countries around the world.^3^,^4^ According to the latest data of Beijing time, May 6, 2020, there were 3,729,072 confirmed cases and 258,622 deaths worldwide. On January 31, 2020, the COVID-19 epidemic requires a coordinated international response, indicating that it may pose risks to multiple countries.^5^ SARS-CoV-2 was isolated from the airway epithelial cells of patients with viral pneumonia in Wuhan.^6^ Compared with known coronaviruses that can infect humans, the SARS-CoV-2 structure has certain differences, so it is defined as the seventh coronavirus.^7^ SARS-CoV-2 belongs to the order Nidovirales, Coronaviridae.^8^ It has an envelope and contains a very large RNA virus genome. The 3D structure of the coronavirus, obtained by cryo-electron tomography, reveals that it is spherical and has an envelope.^9^ Coronavirus is characterized by spikes sticking out from the surface, which in part have the same characteristics as SARS-CoV, and other four Human coronaviruses HCoV-NL63 (human coronavirus NL63), HCoV-229E (human coronavirus 229E), HCoV-OC43 (human coronavirus OC43), and HCoV-HKU1 (human coronavirus HKU1), which only cause mild respiratory diseases.^6^,^8^,^10^ The genome and subgenome of the new coronavirus contain at least 6 open reading frames (ORFs), which generally have 5' leader and 3' end sequences. The 5' cap open reading frame encodes a variety of non-structural proteins. Non-structural proteins are involved in the transcription and replication of the virus. Coronaviruses have at least four major structural proteins, including spikes (S), membranes (M), envelopes (E), and nucleocapsid (N) proteins. Structural proteins are all encoded by the 3' terminus of the viral genome. They are essential for virus-cell receptor binding and the production of structurally complete virus particles.^11^,^12^ Importantly, homology modeling shows that the SARS-CoV-2 binding domain to the ACE2 receptor is structurally similar to SARS-CoV.^13^,^14^ Despite the presence of amino acid mutations in its receptor binding domain, multiple key amino acids are changed.^13^,^14^ However, the amino acid perfectly maintains the stability of the mutual structural conformation of the virus S-protein and the ACE2 receptor in a holistic manner. Preliminary revealed virus uses ACE2 receptor to enter the cell.^13^,^14^

So far, there are no specific drugs specifically used to prevent and treat coronavirus. If there are no vaccine and/or specific antiviral drugs during a virus outbreak, non-specific therapeutic interventions are often used to prevent acute complications and reduce severe morbidity and mortality, such as providing supportive care, including adequate rest, rehydration and analgesics.^15^ At the same time, traditional Chinese medicine, hormonal drugs, broad-spectrum antibiotics, antivirals, antifungals and interferon-α2b can be utilized to minimize the risk of co-infection.^16^

In addition, there is no direct clinical evidence which has shown specific anti- SARS-CoV-2 efficacy, and further substantial research is needed to pay attention to related adverse reactions.

## Drug research based on virus

### Inhibitors of nucleic acid synthesis

Ribavirin (ribavirin) is a synthetic nucleoside antiviral agent with broad-spectrum antiviral activity and inhibits both DNA and RNA viruses.^17^ It is usually used in aerosolized form for adults and children in treatment of respiratory syncytial virus pneumonia.^18^ Early *in vitro* studies have shown that administration of ribavirin can enhance the *in vitro* activity of interferon.^16^ Ribavirin has anti-MERS-CoV activity *in vitro* when used alone or in combination with interferon alpha.^19^ Clinical studies have shown that ribavirin and regulated interferon alpha-2a treatment can significantly improve the 14-day survival rate and slightly improve the 28-day survival rate in patients with MERS-CoV infection.^16^ The timing of initiation of antiviral therapy is critical to the treatment of most patients with viral infections. Adults: 500 mg/time, intravenous infusion, 2 to 3 times a day, the course of treatment does not exceed 10 days.^20^ However, the side effects of ribavirin limit its use to some extent. The use of high-dose ribavirin may be related to hemolytic anemia, neutropenia, teratogenicity, and cardiopulmonary distress.^18^ In view of the curative effect of ribavirin in the treatment of diseases caused by SARS-CoV and MERS-CoV,^21^ it is expected to become one of the effective drugs to treat coronavirus.

Redesivir (RDV, GS-5734), a nucleoside analogue, is a drug under investigation, it has not been approved for marketing in any country yet.^22^ It can exert therapeutic effects by inhibiting the synthesis of viral nucleic acids and has antiviral activity.^23^ Gilead Sciences, Inc. believes that antiviral nucleic acid analogs, such as ribavirin, will be cut out by the coronavirus exonuclease ExoN when integrated into viral RNA during the treatment of coronavirus infection, but RDV is resistant to ExoN. The resistance results in RDV treatment of coronavirus are more effective than other nucleic acid drugs. Previously, RDV was mainly used as a test drug against Ebola virus, and it has a strong anti-filovirus efficacy *in vitro*.^24^ *In vitro* tests, RDV can effectively inhibit the activity of SARS-CoV and MERS-CoV.^23^ For both MERS-CoV and SARS-CoV, its half effective concentration (EC50) is 0.07 μmol/L. In contrast, lopinavir-ritonavir EC50 values ​​are respectively 8 μmol/L and 17 μmol/L.^25^ However, as an effective potential drug for SARS-CoV-2, RDV takes an emergency approach after weighing the risks and benefits. On February 3, 2020, Beijing China-Japan Friendship Hospital led two independent random, double-blind, controlled clinical trials, one for patients with new-type coronavirus mild-to-moderate pneumonia in hospitalized adults (308 cases), and one for patients with severe coronavirus-infected adults (453 cases), to verify the efficacy and safety of ribavirin. The experiments are currently undergoing.

Lopinavir and ritonavir (Kaletra/Aluvia) is the first-line drug for the clinical treatment of AIDS.^26^,^27^ Developed by Abbott, marketed in 2005, mainly combined with viral protease to inhibit protease function. Lopinavir-ritonavir is a compound tablet consisting of lopinavir and ritonavir. Lopinavir is a sensitive substrate for cytochromes CYP3A4 and P-glycoprotein.^26^ It can block the division of Gag-Pol polyprotein and has a high protein binding rate in plasma. Ritonavir is a substrate of CYP3A4, P-glycoprotein and CYP2D6, which inhibit HIV protease: enzymes cannot break down the precursor of Gag-Pol polyprotein. Ritonavir can inhibit CYP3A-mediated lopinavir metabolism, resulting in higher lopinavir concentrations.^26^ *In vitro* studies showed that lopinavir and ribavirin can inhibit the replication of MERS-CoV and SARS-CoV.^28^ Adults: 400 mg/100 mg each time, orally, bid, and the course of treatment does not exceed 10 days.^20^

Darunavir (Prezista) is a second-generation HIV-1 protease inhibitor. It was first marketed in the United States in July 2006. It was developed by Tibotec, a subsidiary of Johnson & Johnson. Darunavir, ritonavir, ritonavir and the combination of other retroviral drugs can be used to treat HIV infection.^29^ It can selectively inhibit the cleavage of HIV-encoded Gag-Pol polyprotein in virally infected cells, thereby inhibiting viral replication.^30^ Darunavir in particular patient population (including pregnant women, pediatrics, patients with HIV-2 infection and co-infection with viral hepatitis) is also safe and effective.^29^

Transmembrane protease serine 2 (TMPRSS2) inhibitors may be used to block SARS-CoV-2 infection and then used to treat COVID-19.^31^ ACE2 is a metal peptidase, expressed on major viral target cells such as lung cells and intestinal epithelial cells, and its catalytic domain binds to the S protein of SARS-CoV with high affinity.^32^ For viral infectivity, host cell proteases affect the S protein cleavage is crucial. TMPRSS2 can activate the spike protein of SARS by lysing the spike protein on the cell surface, which in turn binds to ACE2 and enters the host cell.^33^ TMPRSS2 is expressed in ACE2-positive cells in the human lung.^34^ It is shown that TMPRSS2 may play an important role in the transmission of SARS-CoV in the human respiratory tract.^33^ So far, multiple studies showed that SARS-CoV-2 is likely to bind to human ACE2 receptors and thus invades the human body.^13^,^14^,^31^ Many SARS-CoV-2 prevention and treatment drugs are screened with the ACE2 receptor as a target. In view of the important role of TMPRSS2 in influenza virus and coronavirus infections, TMPRSS2 inhibitors might be useful for clinical treatment.^35^

### RNA polymerase inhibitors

Favipiravir (Avigan) is a broad-spectrum antiviral drug that selectively inhibits the RNA polymerase of influenza viruses.^36^ It was approved for marketing in Japan in March 2014 and it is used for antiviral treatment of influenza A and B.^37^ It is converted into an active phosphoribosylated form in the cell and recognized as a substrate by viral RNA polymerase.^38^ Favipiravir is active against a variety of influenza viruses including A (H1N1) pdm09, A (H5N1), and A (H7N9) avian influenza viruses, and has a synergistic effect with oseltamivir.^37^ In addition to its anti-influenza activity, favipiravir blocks the replication of many other RNA viruses, including alphaviruses, flaviviruses (yellow fever and West Nile virus), enteroviruses (poliomyelitis virus and rhinovirus), Influenza A virus, respiratory syncytial virus and norovirus.^37^,^39^ A number of marketed drugs with antiviral activity, such as favipiravir, were discovered, and follow-up studies need further verification by animal experiments and clinical trials.

### Membrane fusion inhibitors

Abidol (Umifenovir) is a synthetic broad-spectrum antiviral compound developed by the USSR Research Center for Medicinal Chemistry, used to prevent and treat human influenza A and B influenza infections and post-influenza complications.^40^ Abidol was used as an anti-influenza virus drug for decades.^41^ Abidol is active against many DNA/RNA and enveloped/non-enveloped viruses and inhibits membrane fusion between virus particles and plasma membrane and between virus particles and endosome membrane by embedding in membrane lipids.^42^ At present, darunavir and abidol are used in patients with pneumonia infected by a novel coronavirus in Zhejiang province.^20^ The follow-up treatment effect needs further verification.

### Complex inhibitors

Resveratrol is a natural compound found in grape seeds, peels and red wine.^43^ Resveratrol inhibits infections caused by multiple pathogens and shows inhibiting effects in a variety of human viruses, including influenza virus, herpes simplex virus, respiratory syncytial virus, HIV-1, varicella-zoster virus, enterovirus-71, human metapneumovirus, human rhinovirus-16, polyoma virus and cytomegalovirus.^44^,^45^ Resveratrol has antiviral activity against respiratory viruses and certain anti-inflammatory effects, so it can be used as an adjuvant treatment for respiratory infections.^44^ Resveratrol has a wide range of antiviral effects by down-regulating inflammatory signal transduction, which is mainly associated with the inhibition of viral replication, viral protein synthesis, gene expression, and nucleic acid synthesis.^44^-^46^

*In vitro* model evaluations show that resveratrol significantly inhibits MERS-CoV virus infection in a dose-dependent manner and prolongs the survival time of infected cells, which may be related to resveratrol's reduction of nucleocapsid (N) protein expression.^46^ Nucleocapsid (N) protein is a multifunctional protein essential for CoV replication.^47^ In addition, resveratrol down-regulates MERS-CoV-induced apoptosis *in vitro*. In the case of continuous administration, resveratrol can inhibit MERS-CoV at lower doses.^46^ Another study shows that resveratrol also has a significant inhibitory effect on the emerging positive sense RNA Chikungunya virus, indicating that resveratrol may become a candidate drug for the treatment of new RNA viruses.^46^

## Drug research based on host

### Interferon and inducer

After a cell is infected by a virus, it can synthesize and secrete a substance that can interfere virus replication and enhance antiviral ability of nearby cells.^48^ This substance is called interferon. Human interferon is subdivided into type I and type II.^49^ The host's inherent interferon response is critical to control viral replication after infection.^50^ Interferon response can be enhanced by recombinant interferon or interferon inducer.^51^

Recombinant interferon α and interferon β can inhibit the replication of SARS-CoV and MERS coronavirus *in vitro* and in animal models.^52^ Among all subtypes, interferon β1b has the best antiviral effect on MERS-CoV.^16^,^53^ Various combinations of interferon alpha or interferon beta with other antivirals, such as ribavirin, lopinavir, and ritonavir were used to treat patients with SARS and MERS. *In vivo* and *in vitro* studies showed that the combination of ribavirin with IFNα may have a synergistic effect.^16^ The "Pneumonitis Diagnosis and Treatment Scheme for New Coronavirus Infection (Trial Version 7)" states that aerosolized interferon alpha can be used as a trial treatment against SARS-CoV-2 virus to improve the virus clearance effect of respiratory mucosa in patients.^20^ Adults: 5 million IU or equivalent dose of interferon alpha each time, add 2 mL of sterile water for injection or normal saline, inhale by atomization, bid.^20^

Polyinosinic acid-polycytidylic acid is a synthetic analog of dsRNA, which can strongly induce type I interferon. However, a recent study showed that the 4a protein of double-stranded RNA-binding domain interacts with polyinosinic acid-polycytidylic acid, thereby inhibiting polyinosinic acid-polycytidylic acid or Sendai virus-induced interferon production,^54^ but experiments show that polycytidylic acid can still significantly reduce MERS-CoV load in BALB/c mice.^51^

Nitroxanide (NTZ) is another effective type I interferon inducer. It is currently used clinically to treat parasitic infections. It was originally marketed in Mexico under the trade name Daxon/Colufas in 1996, then listed in the United States under the trade name ALINIA in 2002. It is a synthetic thiazolyl-salicylic acid derivative. In cell culture assays, nitrazine can inhibit the replication of a variety of RNA and DNA viruses, including respiratory syncytial virus, parainfluenza virus, and coronavirus.^51^ Nitroxanide has a broad-spectrum antiviral activity: Rotavirus, norovirus, HBV, HCV, dengue virus, yellow fever, Japanese encephalitis virus.^55^ It has been evaluated in phase II and phase III clinical trials for the treatment of HCV infection and the treatment of influenza.^55^,^56^

### Cyclophilin inhibitors

Cyclophilins (cyclophilins, Cyps) belong to the peptidyl-prolyl isomerases (PPIase) family, which are involved in the replication of RNA viruses, including human immunodeficiency virus 1, hepatitis C virus and influenza virus.^57^ Cyclophilin A is the main cellular target of the immunosuppressive drug cyclosporin A (CsA).^57^ CsA is a classic immunosuppressive drug. It binds to the cell cyclophilin to inhibit calcineurin, thereby preventing the translocation of the nuclear factor of activated T cells from to the nucleus, interleukin2 and the transcription of genes.^58^ CsA can inhibit the replication of almost all species of coronavirus in a dose-dependent manner *in vitro*.^59^ Cell culture experiments indicated that CsA strongly inhibited the replication of SARS-CoV, MERS-CoV, human coronavirus 229E, feline coronavirus, avian infectious bronchitis virus, and mouse hepatitis virus, but they showed a significant blocking effect only in the early stages of replication.^59^ Compared to other RNA viruses (0.5-3 µM), a higher CsA concentration (16 µM) is required to block coronavirus replication, which indicates that the coronavirus is less sensitive to CsA treatment.^60^ As an effective and broad-spectrum coronavirus inhibitor, CsA and its analogs have good research and development prospects.^59^

### Glycosylation inhibitors

Chloroquine phosphate is a widely used antimalarial and autoimmune disease drug and has recently been reported as a potential broad-spectrum antiviral drug.^61^,^62^ Chloroquine phosphate can block virus infection by up-regulating the pH of endosomes, required low for virus-cell fusion, and inhibiting glycosylation of cellular receptors. It specifically interacts with sugar-modifying enzymes or glycosyltransferases in human cells.^61^ It has been demonstrated that chloroquine phosphate may have an inhibitory effect on quinone reductase 2, which is structurally adjacent to UDP-N-acetylglucosamine 2-epi-isomerase, thus affecting the biosynthesis of sialic acid.^61^ The presence of a sialic acid moiety in the coronavirus receptor ACE2 may explain the inhibitory effect of chloroquine phosphate on SARS-CoV replication and other functions.^61^,^62^ Recent cellular experiments showed that chloroquine phosphate is very effective in controlling SARS-CoV-2 infection *in vitro*, and it plays a role in the entry phase and post-entry phase of SARS-CoV-2 infection in Vero E6 cells.^56^ In addition to its antiviral activity, chloroquine phosphate has immunomodulatory activity and can synergistically enhance its antiviral effect *in vivo*.^63^ Given the above characteristics of chloroquine phosphate, it is likely to be clinically applicable to SARS-CoV-2.

### Endocytosis inhibitors

Chlorpromazine is an antipsychotic used to treat schizophrenia.^51^ In 1952, chlorpromazine was marketed in France under the trade name Largactil; in 1957, it was approved by the FDA to be marketed under the trade name Thorazine. After most viruses attach to host surface receptors, they enter cells using endocytosis mechanisms (catenin-dependent and independent pathways). SARS uses clathrin-dependent mechanisms to enter host cells.^64^ It has been revealed that chlorpromazine is a broad-spectrum virus inhibitor that can inhibit HCV, alpha virus, and various coronaviruses including human coronavirus 229E, SARS-CoV and MERS-CoV *in vitro*.^25^

### Kinase inhibitors

Imatinib (Gleevec) is a small molecule inhibitor. Nobartis was first marketed in the United States in 2001. It is clinically used to treat chronic myeloid leukemia and malignant gastrointestinal stromal tumors. Imatinib is specifically designed to target Abelson tyrosine-protein kinase 2. Abl2 kinase is a non-receptor tyrosine kinase that exists in the nucleus and mitochondria, and mediates a wide range from embryonic morphogenesis to viral infection cellular processes.^65^ Abl2 kinase is required for SARS-CoV and MERS-CoV to replicate *in vitro*.^66^ As an *in vitro* inhibitor of SARS-CoV and MERS-CoV, imatinib can significantly reduce the titers and inhibit their development process.^66^ Imatinib's anti-coronavirus activity was mainly manifested in the early stages of infection and worked by inhibiting the fusion of viral particles on the endosomal membrane.^66^

## Vaccines and antibodies

### Monoclonal antibodies

Monoclonal antibodies (mAb) were successfully used to treat various diseases.^67^ Passive immunotherapy using neutralizing monoclonal antibodies (mAb) is an effective preventive and therapeutic agent for emerging viruses.^68^ SARS-CoV and MERS-CoV have always been a global public health threat. To date, no vaccines or specific therapies have been approved for diseases caused by these two viruses.^68^

Neutralizing antibodies are a major component of protective immunity against human viral infections. *In vivo* antibody responses mobilize a mixture of dynamic mAbs that work in concert to target various antigens on viral envelope glycoproteins.^69^ Neutralizing mAbs can be achieved by a variety of techniques, such as hybridoma technology, humanized mice, phage or yeast, and single B cell isolation.^67^ More and more mAbs were developed, with high efficacy against emerging viruses *in vitro* and in infected animal models.^67^

Spike glycoproteins play a key role in mediating virus entry and have the capacity to induce protective antibody responses in infected individuals. Consistently with it, spike proteins are the main targets of neutralizing antibodies.^70^ Regeneron et al. used VelocImmune mice to generate fully human non-competitive monoclonal antibodies that bind to MERS-CoV spike protein, and the characterization of two lead monoclonal antibody candidates (REGN 3051 and REGN 3048) were performed *in vitro* and *in vivo*.^70^ Both mAbs effectively neutralize MERS-CoV *in vitro*. Moreover, *in vivo* studies in infected mouse models showed that treatment of infected mice can decrease lung virus titers.^70^ Currently, multiple mAbs for SARS-CoV and MERS-CoV are undergoing the phase Ⅰ clinical trial, which has promising prospects for the treatment of new viral diseases.

Based on the above studies, neutralizing antibodies to spike proteins on the surface of SARS-CoV-2 may be the first therapy considered by researchers.^71^ Recently, GenBank has released the SARS-CoV-2 genomic sequence (MN908947.3). Spike protein can be used as an immunogen to screen mice or rabbits for neutralizing antibodies. However, the development of this traditional method is slow.

Many time-saving methods have been developed, such as the rapid identification of viral neutralization lead candidate phages or yeast display libraries by expressing fragments of antibodies, and their application to the screening of neutralizing antibodies.^72^ On March 4, 2020, a new virus fusion research team (CEVI fusion research team) led by the Korea Chemical Research Institute found antibodies that can fight against SARS-CoV-2. The structure of the spike protein of the SARS-CoV-2 is very similar to that of Middle Respiratory Syndrome (MERS) and SARS, and it was confirmed that the latter can be combined with its neutralizing antibody.^73^

### Serum of rehabilitation patients

Blood-derived immunotherapy based on recovered patients can be used to treat infections including measles virus, Lhasa virus, SARS coronavirus and influenza A H5N1 virus.^74^ This method is also applicable to the treatment of SARS-CoV-2. Rehabilitated patients with new coronavirus pneumonia will produce high-titer polyclonal antibody immune responses to different antigens of SARS-CoV-2. Some of these polyclonal antibodies may neutralize the virus and prevent a new round of infection.^75^ This can be achieved by donating plasma and whole blood into infected patients.^76^ As of today there are more patients with new coronavirus pneumonia in rehabilitation, and plasma can be provided for the treatment of infections if necessary.

## Chinese herbal medicine

On January 27, 2020, the State Administration of Traditional Chinese Medicine conducted clinical observations of the "Qingfei Paidu Decoction" in Shanxi, Hebei, Heilongjiang, and Shaanxi provinces. By February 5, 214 confirmed cases have been treated. The total effective rate has reached more than 90%. On February 6, it began to be promoted and used in China.^77^ The main drug composition of the prescription is "Ephedra 9 g, Roasted Licorice 6 g, Almond 9 g, Raw Gypsum 15 ~ 30 g (first fried), Guizhi 9 g, Alisma 9 g, Polyporus 9 g, Atractylodes 9 g, Poria 15 g, Bupleurum 16 g, Scutellaria baicalensis 6 g, ginger pinellia 9 g, ginger 9 g, aster 9 g, winter flower 9 g, shegan 9 g, asarum 6 g, yam 12 g, citrus aurantium 6 g, tangerine peel 6 g, patchouli 9 g".^20^ The prescription can be used to treat SCoV-infected pneumonia patients with light, normal, and severe types, and can also be used according to actual conditions in the treatment of critically ill patients.

## Challenges in the development of anti-SARS-CoV-2 drugs

With the spread of the COVID-19 epidemic, scientists around the world are actively exploring potentially effective drugs to fight against new coronaviruses, especially RDV for injection. After evaluating the risks and benefits, on February 6, 2020, active treatment was taken in the Wuhan epidemic area, and a phase III clinical trial was conducted, which brought dawn to the clinical treatment of COVID-19. However, there are many difficulties in the development of coronavirus drugs, which restrict the development and application of drugs. Firstly, coronaviruses are RNA viruses with variability, and this is the reason why new types of coronaviruses with novel structures easily appear. The drugs used in the past may not be effective for this new type of coronavirus or have only weak effects. Secondly, many drugs have high EC50/cmax ratios in the clinic and are prone to severe side effects. For example, the use of high-dose ribavirin may be related to hemolytic anemia, neutropenia, and cardiopulmonary distress.^78^ In addition, virus research is highly risky, and general experimental conditions are difficult to meet biosafety requirements, so screening techniques, animal models, and suitable animal experimental platforms are limited. Moreover, the severe epidemic caused by coronavirus has the characteristics of timeliness. There are also some difficulties in clinical trial resources and recruitment of patients with related diseases into clinical trials. Although research of these coronaviruses are based on the results of genome sequencing and bioinformatics, some screening models such as SARS and MERS virus-infected cells, some small mechanisms based on different mechanisms of action such as nucleic acid synthesis inhibitors, protease inhibitors, and polymerase inhibitors have been established. Since low specificity of research and exploration, there is no clinical evidence to show the efficacy of broad-spectrum antiviral drugs against coronavirus. Further substantial research is needed and attention should be given to certain toxic side effects.^18^ The most effective way to fight against the virus remains the vaccine. Compared with the long cycle required for drug development, the vaccine takes a relatively short time and has a strong protective effect. Monoclonal antibodies have better effects, stronger specificity, and higher safety, but they also require more time and cost to develop.

Compared to SARS and MERS, humans have made great progress in understanding the new coronavirus, the mechanism of drug action, research and development strategies, both in terms of knowledge and technology. In the waiting period for the development of new coronaviruses, China is now using multiple drugs to treat new coronaviruses. Although there are many risks, it is undeniably the most effective approach at present.

## Conclusion

According to Chinese anti-SARS-CoV-2 diagnosis and treatment program and treatment experience, a combination of multiple drugs is adopted, but it is not recommended to use 3 or more antiviral drugs (Table 1)^20^. This will alleviate the symptoms of the patient and enhance the patient's immunity, thereby achieving the purpose of treating the disease.

## Figures and Tables

**Table 1:** The commonly used anti-COVID-19 drug index in China.

Drugs	Categories	Pharmacological effects	Drug types	Adult treatment usages and dosages	Administration time	The elderly	Warning
Lopinavir/ Ritonavir	Protease inhibitors	Inhibit the function of protease, block the division of gag-pol polyprotein	Tablet	400/100 mg, bid	Regardless of diet	Elderly use with caution	Do not break apart or crush
Abidol (Umifenovir)	Membrane fusion inhibitor	Block viral replication by inhibiting the fusion of influenza virus lipid membranes with host cells	Tablet	200 mg, po, tid, 5 d	Regardless of diet	The safety of medication for the elderly over 65 years old is not yet clear	Nothing
Ribavirin	Inhibitors of nucleic acid synthesis	Inhibit the synthesis of viral RNA and protein by inhibiting intracellular synthesis of guanosine triphosphate	Injection	500 mg, iv, bid or tid, treatment course does not exceed 10 d	Regardless of diet	Not recommended for the elderly	Nothing
Chloroquine	Glycosylation inhibitors	Prevents replication and transcription by forming a complex with DNA	Tablet	500 mg, bid or tid, treatment course 7 d	Regardless of diet	Security is not clear	Nothing
α-Interferon	Cytokines	Regulates human immunity	Injection	5 million IU, fog, bid	Regardless of diet	Elderly patients should reduce the dosage	Nothing

These data are from the seventh edition of China's anti-novel coronavirus pneumonia diagnosis and treatment plan.
